# Prevalence and genotype distribution of HPV infections among women in Chengdu,China

**DOI:** 10.1186/s12985-024-02317-x

**Published:** 2024-03-01

**Authors:** Junying Zhang, Tianzhi Zha, Xuemei Wang, Weijun He

**Affiliations:** 1 Clinical Laboratory Department, Chengdu Women’s and Children’s Central Hospital, School of Medicine, University of Electronic Science and Technology of China, Chengdu, 611731 China; 2 Chengdu Angel medical equipment Co., LTD, Chengdu, China

**Keywords:** Human papillomavirus, Genotype, Cervical cancer, HPV vaccine

## Abstract

**Background:**

Human papilloma virus (HPV) infection among female is the cause of cervical cancer and genital warts. In China, the HPV vaccination rate and the target population screening rate among females are low, and the aims of this study on the genotype distribution and prevalence of HPV infection were to provide more targeted strategies for the prevention and treatment of cervical cancer and HPV-related diseases.

**Methods:**

Polymerase chain reaction-reverse dot blot (PCR-RDB) was adopted for HPV genotyping test, the prevalence and 23 genotypes distribution of HPV infections among 181,705 women in Chengdu from 2013 to 2020 were analysed.

**Results:**

The overall prevalence rate of HPV infection among 181,705 cases was 23.28%, the prevalence of HR-HPV at the age group < 20 years, 60–69 years and ≥ 70 years were higher than the overall prevalence.The prevalence of HPV showed a bimodal U-shaped curve with age; the first and second peak common occurred among females < 20 years old (42.97%) and 60–69 years old (37.56%), respectively.The top five genotypes of HPV infection among females in Chengdu were HPV52/16/58/81/53. Single infection (73.26%) was the main HPV infection pattern, followed by double infection (19.17%) and multiple infection (7.57%), the infection rate of HPV showed a gradual declined as the patterns of HPV coinfections increased, low-risk and high-risk coinfection was higher in low-risk HPV infection (43.68%) and lower in high-risk HPV infection (13.59%). The prevalence of genotypes − 6 and − 81 infection was the second highest at the age group of 20 and 40–59, respectively, while the prevalence of HPV16 was the highest at the age group of ≥ 70 among 23 genotypes among the 181,705 women.

**Conclusions:**

The prevalence of HPV infections among women in Chengdu is higher than domestic certain developed citys, among the five vaccines available, nonavalent vaccine is more suitable for Chengdu females. For young females prioritizing vaccination is essential in the current context.Double screening for HPV DNA is important in middle-aged women (30–49 years), and screening should not be lacking in older women (> 65 years). Additionally,for patients with genital warts, it is necessary to screen for high-risk HPV infection and provide appropriate management and treatment. Given the limitations of this study, future HPV research should aim to achieve full coverage of the target population, and our studies should also include cellular or pathological data of HPV-positive cases, vaccination rates, and various lifestyle details.

## Introduction

Human papillomaviruses (HPVs) are non-enveloped, double-stranded circular DNA viruses of 8 kb in size that are primarily transmitted through having vaginal, anal, or oral sex with someone who has the virus, even if they don’t have signs or symptoms, and which cause epithelial hyperplasia of the skin and other diseases (cervical cancer, oropharyngeal cancers, anal cancer, precancers and genital warts) [1]. HPVs belong to the Papovaviridae family and humans are the sole host. The HPVs that infect the mucosal epithelium are defined as Alphapapillomaviruses [2]. To date, more than 200 types of HPV have been identified, more than 40 of which infect the genital area. Based on their carcinogenicity, mucosal alpha-HPV is classified as high-risk HPV and low-risk HPV (HR-HPV and LR-HPV). HR-HPV is closely related to the development of malignant carcinomas, and LR-HPV mainly causes benign warts. 15 HPV types were classified as HR-HPVs (16, 18, 31, 33, 35, 39, 45, 51, 52, 56, 58, 59, 68, 73, and 82), 12 HPV types were classified as LR-HPVs (6, 11, 40, 42, 43, 44, 54, 61, 70, 72, 81, and CP6108) [3]. Based on HPV activities, HPV genome is classified into three regions: the long control region (LCR) or the non-coding upstream regulatory region (URR), the late (L) region, and the early (E) region,among them the E region encodes six early regulatory proteins (E1, E2, E4, E5, E6, and E7) and the L region encodes structural proteins (L1 and L2), which have different functions that E1 and E2 modulate transcription and replication, E5, E6, and E7 modulate the transformation process andL1 and L2 compose the viral icosahedral capsid [4]. HPV infections are very common, acquisition of new partners and having had more than 1 sexual partner were strong determinants of new infections [5]. However, most HPV infections are transient, with automatic clearance of 60% and more than 90% at one and two years [6], moreover, only a small minority of persistent oncogenic HPV infection progress to cervical intraepithelial neoplasia (CIN), precancerous lesions, and cancer. One of the mechanisms HPV causes cancer is that the virus prevents cell cycle arrest and apoptosis,the virus E6 and E7 oncoproteins inactivate the tumor suppressor proteins p53 and pRB, respectively, resulting in uncontrolled cell proliferation and the transfer of their genetic material into the nucleus of the target cell another is the integration of HPV DNA into the host genome [7, 8]. Approximately 98.7% of cervical cancers are attributable to HR-HPV [9]. HPV 16 and HPV18 are responsible for approximately 71% of cervical cancer cases [10].

According to the Global Cancer Statistics in 2020 [11], cervical cancer is the fourth most frequently diagnosed cancer and the fourth leading cause of cancer death in global women, with an estimated 604,000 new cases and 342,000 deaths worldwide. Cervical cancer is a major public health problem that threatens the lives of women worldwide, and the vast majority of cervical cancers are found in countries such as Sub-Saharan Africa, Melanesia, South America and Southeast Asia. Nearly 70% of the global burden of cervical cancer occurs in less developed countries. Currently, the elimination of cervical cancer is a major public health issue worldwide. As a developing country with the larger population in the world, China bears a serious burden of cervical cancer. According to the latest statistics from the ICO/IARC HPV Information Centre in 2023 [12], China experiences approximately 109,741 new cases and 59,060 deaths related to cervical cancer annually.

LR-HPV primarily causes genital warts and recurrent respiratory papillomatosi, 90% of which are caused by noncarcinogenic LR-HPV 6 or/and 11, and which are estimated to affect 1% of sexually active adults aged between 15 and 49 [13]. Although genital warts are benign, they can cause discomfort and affect mental health, even with liquid nitrogen cryotherapy or freeze the probe, they cannot be eradicated due to HPV infection can lead to recurrence under external stimulation, which poses considerable challenges for treatment.

The most effective approach for controlling cervical cancer and genital warts is prioritizing HPV vaccination.There are three types of HPV vaccines, bivalent (16, 18), quadrivalent (6, 11, 16, 18) and nonavalent vaccine (6, 11, 16, 18, 31, 33, 45, 52, and 58), all of which have been proven to have good efficacy, longer-lasting protective effectiveness and benefits [14–16]. A study of HPV vaccination in 179 countries [17] noted that vaccination of a cohort of 58 million 12-year-old girls prevented 690 000 cases of cervical cancer and 420 000 deaths during their lifetime. In the United States [18] HPV infections have dropped 71% among teen girls and 61% among young adult women. Due to various factors, the HPV vaccine was introduced to Chinese females a decade after their global counterparts. Currently, the China Food and Drug Administration (CFDA) has approved a total of five vaccines to be marketed in mainland China, including three vaccines that are available internationally (Cervarix®2, Gardasil®4, Gardasil®9) and two locally produced bivalent vaccines (16, 18) (Cecolin®approved in 2019 and 沃泽惠®approved in 2022). Owing to economic constraints, the HPV vaccine is only promoted as a national secondary vaccine. Nationally, HPV vaccine coverage is less than 0.05% among eligible women ages 9–45, and women ages 9–14 account for less than 5% of the vaccinated population [19].

Regular cervical cancer screening is another strategy to prevent cervical cancer. To eliminate cervical cancer in women globally, WHO [20] calls for that 70% of women are screened with a high-performance test by 35 years of age and again by 45 years of age by 2030. HPV Test is the most sensitive among all the screening tests available till date,among women who test negative on an HPV test, rescreening should be done after a minimum interval of five years [21], therefore,if resources permit, a validated HPV testing for oncogenic HPV types should be the first choice test. In China, the screening rate of women aged 35–44 in 2018–2019 was 43.4% [22], which was lower than the 70% screening rate target proposed by the WHO, moreover, HPV DNA screening of the target population is very limited, and most of them are organized by institutions in hospitals.

In view of the high incidence of cervical cancer in Chinese women, the delayed vaccination and low vaccination rate, and the low screening rate of the target population, and the existing literature lacks large-scale epidemiological studies specifically focusing on the western region of China, and this study was based on the substantial dataset of 181,705 cases of female HPV screening in a large women’s and children’s specialized hospital in Chengdu, and the data came from one central institution and three branches, covering almost the entire urban and suburban areas of Chengdu, which had certain representability of female HPV infection in Chengdu, and the objective of this study were to provide more targeted strategies for the prevention, treatment and elimination of cervical cancer and HPV-related diseases. In this study, we conducted a comprehensive and in-depth exploration of the patterns of HPV infection using real-world data, specifically addressing three aspects: age-specific HPV infection, type-specific and year-specific HPV infection. The content of this study is thorough and holds significant implications for guiding clinical practice.

## Materials and methods

### Subjects

The methodology of this research was a retrospective study covering the period from June 2013 to September 2020, and the subjects were the patients who came from Chengdu Women’s and Children’s Central Hospital (CWCCH) for HPV screening motivated by a variety of reasons, including physical examination, patient requests, diagnostic needs prescribed by doctors and opportunistic screening by doctors. Specific reasons included health examinations, abnormal vaginal bleeding, lower genital tract inflammation, genital warts, infertility, unknown lower abdominal pain, gynaecological tumours, and urethritis. Inclusion criteria consisted of a history of sexual activity, absence from menstruation and pregnancy, while exclusion criteria included women with no history of sexual activity, menstruating and pregnant women, women who had undergone uterine surgery. For the multiple cases, we only counted the results of the first screening. A total of 16,100 cases were excluded from the study. Finally, a total of 181,705 females were included in this retrospective study conducted at CWCCH. The mean age was 34.06 ± 7.8 years (ranging from 1 to 91 years),categorized into age groups < 20, 20–29, 30–39, 40–49, 50–59, 60–69 and ≥ 70. This study was approved by the Medical Ethics Committee of Chengdu Women’s and Children’s Central Hospital, and all methods were performed in accordance with the relevant guidelines and regulations.

### Specimen collection

A gynaecologist wipes cervical secretions with a cotton swab before sampling, places the cervical brush head on the cervix and rotates the brush head clockwise 5 times to obtain a sufficient amount of cervical epithelial cells. Then, the cervical brush head is placed into the sample tube marked with the patient’s name, the cap is tightened, and the sample is quickly sent for examination.

### DNA extraction and HPV genotyping

DNA extraction and HPV genotyping were performed using an HPV genotyping kit for 23 Types (Shenzhen Yaneng Bio-Tech Co., Ltd.) by PCR-RDB which was a cost-effective and beneficial cervical cancer primary screening for hospital-based opportunistic screening [23]. The kit detected 23 types of HPV: HPV6, 11, 42, 43, 16, 18, 31,33, 35, 39, 45, 51, 52, 53,56, 58, 59, 66, 68, 73,81,82,83. The process involved three steps: HPV-DNA extraction, PCR amplification and HPV-DNA hybridization. The Haema9600 (Zhuhai XZ Bio-Tech Co., Ltd.) was used for gene amplification. The amplification parameters were set as follows: ①50 ℃ for 15 min; ②95 ℃ for 10 min; ③40 cycles were performed at 94 ℃ for 30 s, 42 ℃ for 90 s and 72 ℃ for 30 s; and ④ 72℃ for 5 min. The YN-H16 (Shenzhen GL Bio-Tech Co., Ltd.) was used for DNA hybridization. After color rendering, positive detection results were indicated by clear blue dots, a blue spot in one gene locus is a single infection, and and multiple blue spots were mixed infection or multiple infectiona. To ensure the reliability of each HPV test result, there is an IC (internal control) site on each patient’s hybrid membrane, with one positive control (HPV16 positive) and one negative control set up during each trial. In this study, in order to exclude the interference of reproductive pathogens other than HPV (CT, MH, TV, TP, SPY, HSH-2, etc.) on HPV detection results, specific reagents were added during DNA extraction, amplification, and hybridization to ensure that there was no cross-reaction.The testing doctor strictly follows the HPV interpretation rules to issue the patient’s HPV test results.

### Statistical analysis

Microsoft’s Excel 2021 was used to count and analyse the data, the chi-square test was used to compare the sample rate between the groups, SPSS 26 (SPSS Inc., Chicago, IL, USA) was used to calculate chi-square data, *P* values and 95% confdence interval (95% CI), and two-sided *P* values of less than 0.05 were considered statistically significant.

## Results

### Overall HPV infection prevalence

**Fig. 1:**
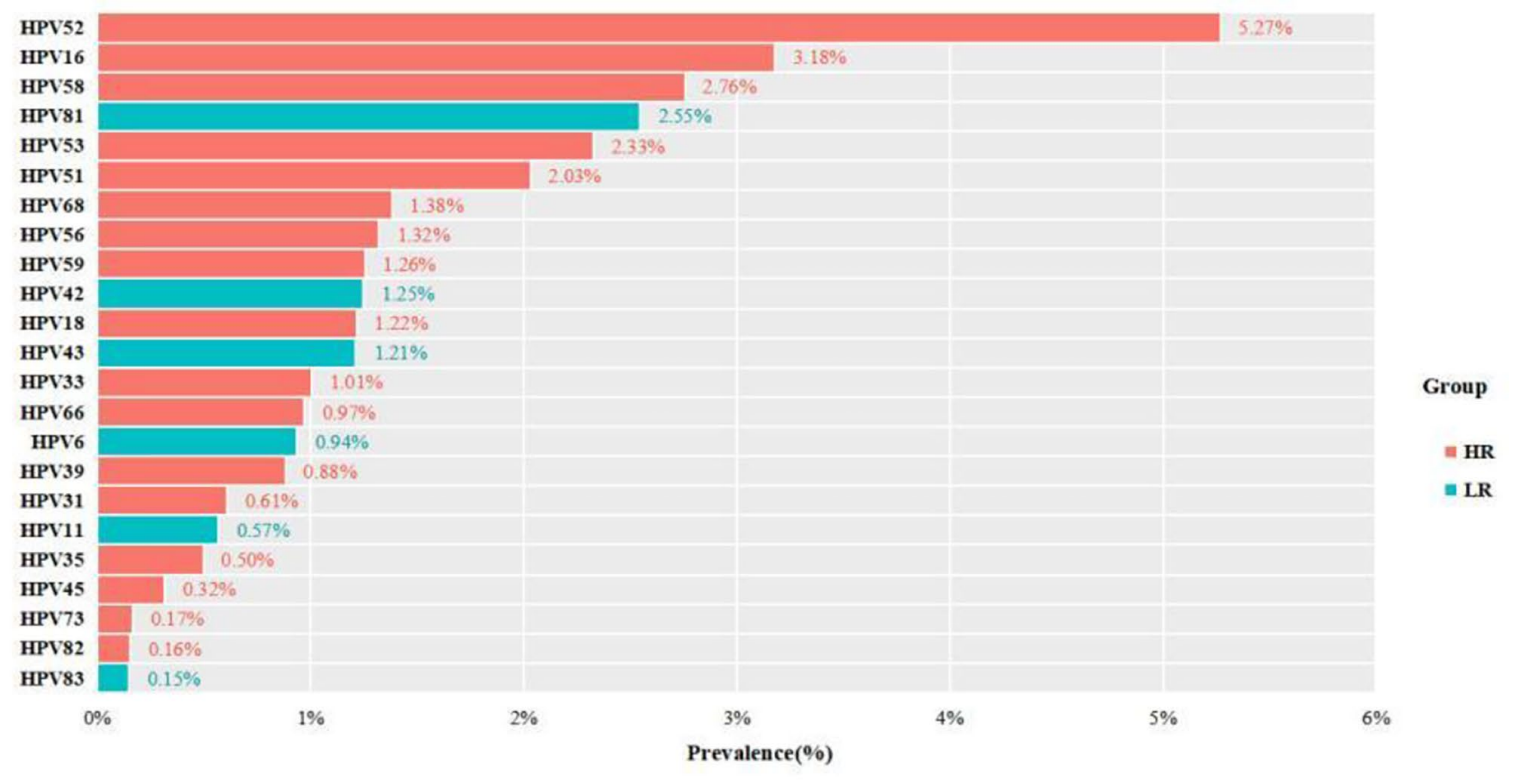
The prevalence and genotype distribution of HPV(HR-HPV and LR-HPV) infections among females from 2013 to 2020

The study involved 23 HPV genotypes (17 HR -HPV and 6 LR- HPV) in 181,705 female cases in Chengdu from 2013 to 2020, and the overall HPV infection positive rate was 23.28% (42,297 positive cases) (Tables 1 and 2). Among 42,297 positive cases,the single infection rate was 17.05% (30,985/181,705), and the multiple infection rate was 6.23% (11,312/181,705), consequently, the single infection was the main pattern of HPV infection among women, and in multiple infections of 11,312 women, the prevalence of HR-HPV and HR-HPV coinfection, HR-HPV and LR-HPV coinfection, and LR-HPV and LR-HPV coinfection was 3.37% (6130 positive cases), 2.69% (4892 positive cases) and 0.16% (290 positive cases), respectively, therefore, the coinfection of HR-HPV and HR-HPV was the highest, followed by the coinfection of HR-HPV and LR-HPV, and the coinfection of LR-HPVand LR-HPV was the lowest (Table 1). Among which the positive rates of HR- HPV only infection cases, LR-HPV only infection cases and HR -and LR- mixed HPV infection cases were 17.11% (31,098 positive cases), 3.47% (6307 positive cases) and 2.69% (4892 positive cases), respectively (Table 2), and it can be seen from this that the HPV infection types of positive cases were mainly HR- HPV only. The positive rate of 23 genotypes was 32.05% (58,233 positive genotypes), among which the positive rate of HR-HPV was 25.38% (46,112 positive) and the positive rate of LR-HPV was 6.67%(12,121 positive) (Tables 3 and 4). The 17 high-risk types were HPV-52,-16,-58,-53,-51,-68, -56, -59,-18,-33,- 66,-39,-31,-35,-45,-73 and − 82 in order, in which the top 5 genotypes were HPV-52,-16,-58,-53 and − 51, with positive rates of 5.27%, 3.18%, 2.76%, 2.33% and 2.03%, respectively. The six low- risk types were HPV − 81,-42, -43, -6, -11 and − 83 in order,with positive rates of 2.55%, 1.25%, 1.21%, 0.94%,0.57% and 0.15%, respectively (Fig. 1; Tables 3 and 4).

### Year-specific prevalence of HPV infection

**Fig. 2:**
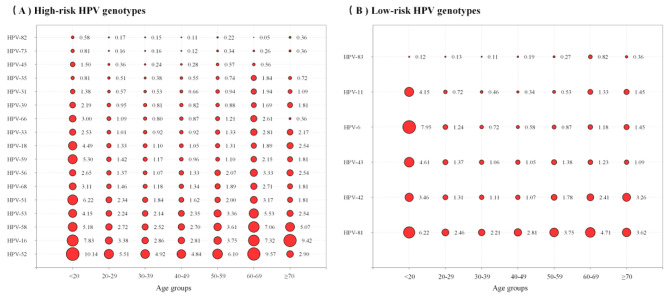
Bubble plots showing the relative prevalence of detected 23 HPV genotypes across age groups. **A** 17 high-risk HPV genotypes. **B** 6 low-risk HPV genotypes

**Fig. 3:**
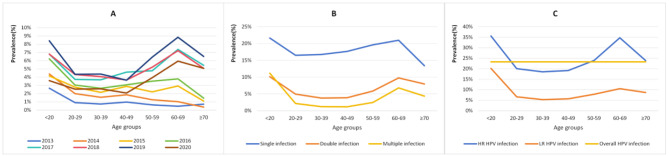
The prevalence of HPV infection by age groups. **A** HPV infections from 2013 to 2020. **B** Single infection. double infection and multiple infection. **C** High-risk, low-risk high-risk and low-risk mixed HPV infection and overall HPV infection

**Fig. 4:**
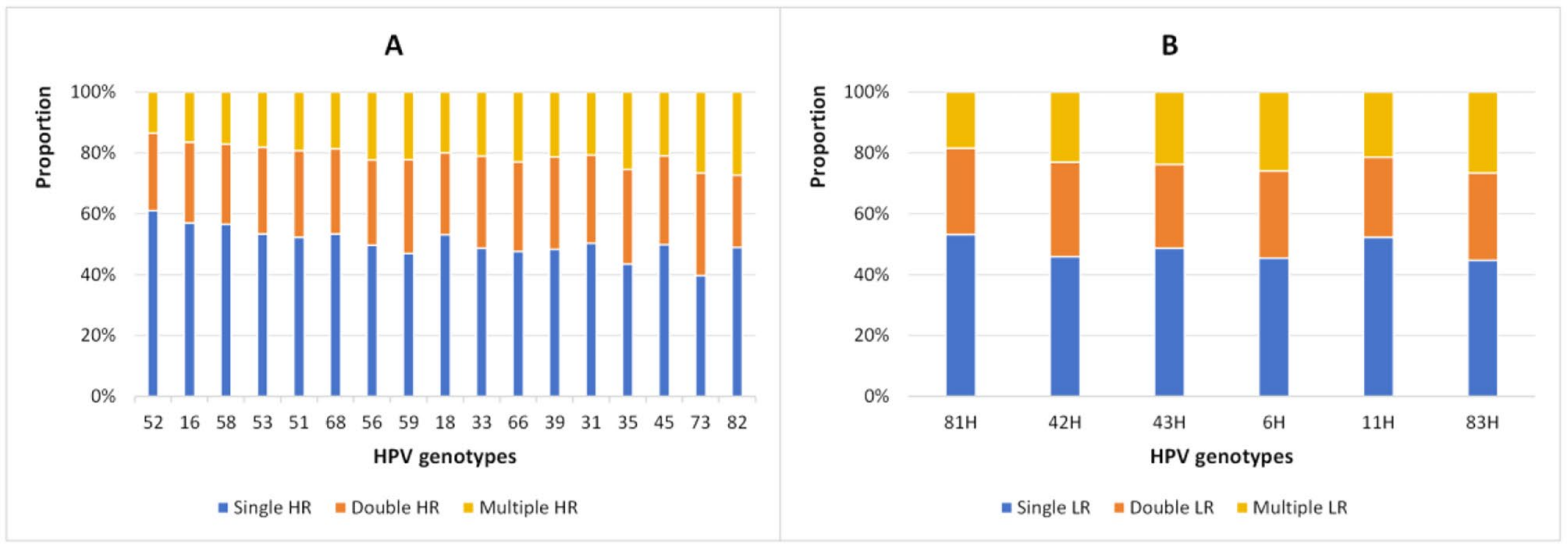
The genotype distribution of single HPV, double HPV and multiple HPV infections among female from 2013 to 2020. **A** HR-HPV. **B** LR-HPV

In HR-HPV infections among 181,705 females from 2013 to 2020, the top five genotypes were common HPV-52,-16,-58,-53 and − 51 in order, whereas LR-HPV infections were common HPV-81,-42,-43,-6,-11 and − 83 in order (Table 3). With the increase in the patterns of multiple infections, the prevalence of multiple infections decreased gradually, and this trend was observed almost every year and in almost every age group from 2013 to 2020 (Tables 1, 2 and 4; Figs. 3B and 4). The age distribution curves from 2013 to 2020 all showed U-shaped, and the first peak (< 20 years) was the highest, but the second peak (40–49 years in 2013–2014, 60–69 years in 2015–2020) was slightly lower (Table 2; Figs. 2 and 3). From 2013 to 2020, the prevalence of the single LR-HPV, HR-HPV and HR-HPV coinfection, HR-HPV and LR-HPV coinfection, infection pattern of single, double, triple, quad, five were significantly different (*p* < 0.05) (Table 1).

**Table 1:** Prevalence of infection patterns and infection types of 23 genotypes from 2013 to 2020

	Positive cases	% (95% CI)	2013 (*n* = 8761)	2014 (*n* = 14,752)	2015 (*n* = 20,669)	2016 (*n* = 24,972)	2017 (*n* = 29,944)	2018 (*n* = 29,065)	2019 (*n* = 33,642)	2020 (*n* = 19,900)	χ^2^	*P*
**Positive cases**	42,297	23.28(23.08–23.47)	1519(17.34)	3190(21.62)	4601(22.26)	5344(21.4)	7228(24.14)	7641(26.29)	8046(23.92)	4728(23.76)	217.219	< 0.001
**Infection types**												
**Single L**	6017	3.31(3.23–3.39)	279(3.18)	526(3.57)	674(3.26)	758(3.04)	1041(3.48)	1028(3.54)	1126(3.35)	585(2.94)	0.529	0.467
**Single H**	24,968	13.74(13.58–13.90)	866(9.88)	1756(11.9)	2596(12.56)	3187(12.76)	4352(14.53)	4585(15.77)	4800(14.27)	2826(14.2)	197.919	< 0.001
**LR + LR**	290	0.16(0.14–0.18)	12(0.14)	23(0.16)	34(0.16)	39(0.16)	59(0.2)	56(0.19)	47(0.14)	20(0.1)	0.816	0.366
**HR + HR**	6130	3.37(3.29–3.46)	196(2.24)	480(3.25)	690(3.34)	760(3.04)	1058(3.53)	1122(3.86)	1114(3.31)	710(3.57)	24.764	< 0.001
**HR + LR**	4892	2.69(2.62–2.77)	166(1.89)	405(2.75)	607(2.94)	600(2.4)	718(2.4)	850(2.92)	959(2.85)	587(2.95)	16.266	< 0.001
**Infection pattern**												
**single**	30,985	17.05(16.88–17.23)	1145(13.07)	2282(15.47)	3270(15.82)	3945(15.8)	5393(18.01)	5613(19.31)	5926(17.61)	3411(17.14)	157.056	< 0.001
**double**	8109	4.46(4.37–4.56)	281(3.21)	631(4.28)	942(4.56)	996(3.99)	1331(4.44)	1480(5.09)	1519(4.52)	929(4.67)	28.806	< 0.001
**triple**	2254	1.24(1.19–1.29)	68(0.78)	199(1.35)	268(1.3)	278(1.11)	359(1.2)	414(1.42)	411(1.22)	257(1.29)	4.273	0.039
**quad**	650	0.36(0.33–0.39)	18(0.21)	55(0.37)	87(0.42)	78(0.31)	97(0.32)	90(0.31)	133(0.4)	92(0.46)	4.333	0.037
**five**	192	0.11(0.09–0.12)	4(0.05)	14(0.09)	16(0.08)	27(0.11)	24(0.08)	31(0.11)	46(0.14)	30(0.15)	9.464	0.002
**≥six**	107	0.06(0.05–0.07)	3(0.03)	9(0.06)	18(0.09)	20(0.08)	24(0.08)	13(0.04)	11(0.03)	9(0.05)	3.603	0.058

### Age-specific prevalence of HPV infection

Among the 181,705 women who participated in HPV screening, the age groups < 20, <30, 20–59, 60–69 and ≥ 70 accounted for the proportion 0.48%, 38.09%, 92.27%, 1.08%, 0.15%, respectively, therefore, HPV infections among the women aged 20–59 are the main age group in this study, and there are fewer cases among the women at other age groups (< 20 years, 60–69 years, ≥70 years) (Table 2).

From 2013 to 2020, the HPV infection curve showed a bimodal U-shaped with age, and the trend was found in every of 23 genotypes, every infection pattern, every infection type and every year (Table 2; Figs. 2 and 3). The first peak occurred < 20 years (42.97%), sharply declining after the first peak and maintaining a plateau during middle age, followed by a slight increase with age, reaching the second minor peak (37.56%), however, the second peak were at age group 40–49 years in 2013–2014 (0.97% in 2013 and 1.85% in 2014, respectively) and were at age group 60–69 years in 2015–2020 (2.92%, 3.79%, 7.37%, 7.22%, 8.85% and 5.94%, respectively, in 2015–2020) (Table 2; Fig. 3A).

The prevalences of single infection, double infection and multiple infection among women also showed a bimodal U-shaped distribution with age,and in which the infection rates of the first peak (< 20 years) were 21.66% (188/868), 10.14% (88/868) and 11.18% (97/868), respectively,and the second peak (60–69 years) was slightly lower than the first peak, with the infection rates of 20.98% (410/1954), 9.77% (191/1954), and 6.81% (133/1954), respectively (Table 2; Fig. 3B). The prevalences of both HR-HPV infection and LR-HPV infection among women showed the same U-shaped distribution, among which the infection rates of the first peak (< 20 years) were 35.60% (309/868) and 20.16% (175/868), respectively, and the infection rates of the second peak (60–69 years) were 34.75% (679/1954) and 10.49% (205/1954), respectively (Table 2; Fig. 3C).

The prevalences of HR-HPV infections at the age groups < 20, 60–69 and ≥ 70 were higher than the overall prevalence (23.28%), with the infection rates of 35.6%, 34.75%, and 23.91% (66/276), respectively (Table 2; Fig. 3C). The prevalences of the HR-HPV infection at the 20–59 age groups and the whole LR-HPV infections at each age group were lower than the overall prevalence 23.28% (Table 2; Fig. 3C). HPV infections in 2013–2015,2017 and 2019–2020, single infection, triple infection and HR-HPV only infection with age were significantly different (*p* < 0.05) (Table 2).

**Table 2:** Prevalence of HPV infection at different age groups among 181,705 females from 2013 to 2020

	Positive cases	% (95% CI) for all samples	< 20(*n* = 868)	20–29(*n* = 68,351)	30–39(*n* = 68,170)	40–49(*n* = 31,130)	50–59(*n* = 10,956)	60–69(*n* = 1954)	≥ 70(*n* = 276)	χ^2^	*P*
Positive cases	42,297	23.28(23.08–23.47)	373(42.97)	16,145(23.62)	14,834(21.76)	7076(22.73)	3064(27.97)	734(37.56)	71(25.72)	43.652	< 0.001
Years											
2013	1519	0.84(0.79–0.88)	23(2.65)	612(0.90)	501(0.73)	303(0.97)	69(0.63)	9(0.46)	2(0.72)	6.851	0.009
2014	3190	1.76(1.70–1.82)	38(4.38)	1360(1.99)	1058(1.55)	576(1.85)	137(1.25)	20(1.02)	1(0.36)	41.036	< 0.001
2015	4601	2.53(2.46–2.60)	36(4.15)	1897(2.78)	1469(2.15)	897(2.88)	242(2.21)	57(2.92)	3(1.09)	5.404	0.02
2016	5344	2.94(2.86–3.02)	54(6.22)	2075(3.04)	1797(2.64)	955(3.07)	385(3.51)	74(3.79)	4(1.45)	1.216	0.27
2017	7228	3.98(3.89–4.07)	59(6.80)	2545(3.72)	2506(3.68)	1435(4.61)	524(4.78)	144(7.37)	15(5.43)	79.103	< 0.001
2018	7641	4.21(4.11–4.30)	59(6.80)	2955(4.32)	2766(4.06)	1135(3.65)	571(5.21)	141(7.22)	14(5.07)	1.684	0.194
2019	8046	4.43(4.33–4.52)	73(8.41)	2968(4.34)	2978(4.37)	1131(3.63)	705(6.43)	173(8.85)	18(6.52)	30.282	< 0.001
2020	4728	2.60(2.53–2.68)	31(3.57)	1733(2.54)	1759(2.58)	644(2.07)	431(3.93)	116(5.94)	14(5.07)	33.384	< 0.001
Infection pattern											
Single	30,985	17.05(16.88–17.23)	188(21.66)	11,282(16.51)	11,417(16.75)	5500(17.67)	2151(19.63)	410(20.98)	37(13.41)	64.695	< 0.001
Double	8109	4.46(4.37–4.56)	88(10.14)	3379(4.94)	2577(3.78)	1208(3.88)	644(5.88)	191(9.77)	22(7.97)	0.374	0.541
triple	2254	1.24(1.19–1.29)	42(4.84)	1051(1.54)	619(0.91)	284(0.91)	181(1.65)	74(3.79)	3(1.09)	8.197	0.004
quad	650	0.36(0.33–0.39)	27(3.11)	297(0.43)	158(0.23)	67(0.22)	60(0.55)	35(1.79)	6(2.17)	0.002	0.966
five	192	0.11(0.09–0.12)	11(1.27)	92(0.13)	51(0.07)	9(0.03)	17(0.16)	12(0.61)	0(0)	3.339	0.068
≥six	107	0.06(0.05–0.07)	17(1.96)	44(0.06)	12(0.02)	8(0.03)	11(0.10)	12(0.61)	3(1.09)	0.356	0.551
Infection types											
HR only^*^	31,098	17.11(16.94–17.29)	198(22.81)	11,610(16.99)	11,203(16.43)	5308(17.05)	2203(20.11)	529(27.07)	47(17.03)	61.149	< 0.001
LR only^**^	6307	3.47(3.39–3.56)	64(7.37)	2429(3.55)	2208(3.24)	1108(3.56)	438(4.00)	55(2.81)	5(1.81)	0.258	0.611
HR + LR	4892	2.69(2.62–2.77)	111(12.79)	2106(3.08)	1423(2.09)	660(2.12)	423(3.86)	150(7.68)	19(6.88)	0.138	0.71

^*^. means single HR-HPV infection and coinfection of HR-HPV and HR-HPV

^**^. means single LR-HPV infection and coinfection of LR-HPV and LR-HPV

### Type-specific prevalence of HPV infection

From 2013 to 2020,the prevalences of 23 genotypes were common HPV-52, -16, -58, -81, -53, -51, -68, -56, -59, -42, -18, -43, -33, -66, -6, -39, -31, -11, -35, -45, -73, -82 and -83 in order (Fig. 1). However, there were some differences in the prevalence rates among genotypes with year, infection pattern and age group. in 2013 and in 2020 the prevalences of the top five HR-HPV genotypes were HPV-52, -16, -58, -33, -53 and HPV-52, -58, -16, -53, -51, respectively, and in 2013–2015, in single infections and multiple infections the prevalence of 6 LR-HPV genotypes were HPV-81, -43, -42, -6, -11, and − 83 in order (Tables 3 and 4), and at the age group < 20 years the prevalence of HPV6 ranked the second (7.95%), whereas at the age groups 40–49 years and 50–59 years the prevalences of HPV-81 and − 16 were tied for the second highest, and the prevalence rates of these two age groups were 2.81% and 3.75%, respectively, and at the age group ≥ 70 years, HPV16 (9.42%) and HPV52 (2.90%) ranked first and fifth, respectively (Fig. 2). From 2013 to 2020, the prevalence of HPV-52, -16, -58, -53, -51, -68, -56, -59, -66, -39, -31, -45, -82, -81, -42, -43 and − 83 were significantly different (*p* < 0.05) (Table 3).

From 2013 to 2020, the prevalences of 23 HPV infections decreased as HPV infection patterns increased each year,among 42,297 HPV positive women in Chengdu, the prevalences of the HPV infection patterns about single, double, triple, quad, five and ≥ six were 17.05%, 4.46%, 1.24%, 0.36%, 0.11% and 0.06%, respectively (Tables 1, 2 and 4; Fig. 4). Among 42,297 HPV positive females, the positive rates of HR-HPV only, LR-HPV only, HR- and LR-HPV mixed infection were 73.52% (31,098/42,297), 14.91% (6307/42,297) and 11.57% (4892/42,297), respectively. Among 35,990 HR -HPV infection women, the positive rate of mixed HR-and LR-HPV infection was 13.59% (4892/35,990), and among 11,199 LR-HPV infection women, the positive rate of LR- and HR-HPV mixed infection was 43.68% (4892/11,199), therefore, the positive rates of HR-and LR-HPV mixed infection in the LR-HPV infection was higher than that of the HR-HPV infection (Tables 1 and 2).

From Table 5, it can be observed that the total proportion of the top 1 to 10 genotypes in combined infections with the other 22 genotypes ranges from 69.54 to 76.41%. The top 1 to 10 genotypes were typically also among the top 10 genotypes of the 23 genotypes. Among the top 1 to 10 genotypes, LR-HPV infection rate was lower, approximately ranging from 13.74 to 27.26%. Therefore, in multiple infections, HR-HPV multiple infections dominate (Table 5).

**Table 3:** Type-specific prevalence of HPV infection from 2013 to 2020

HPV subtype	Positive cases	% (95% CI) for all samples	2013(*n* = 8761)	2014(*n* = 14,752)	2015(*n* = 20,669)	2016(*n* = 24,972)	2017(*n* = 29,944)	2018(*n* = 29,065)	2019(*n* = 33,642)	2020(*n* = 19,900)	χ^2^	*P*
**52**	9580	5.27(5.17–5.38)	306(3.49)	782(5.3)	1100(5.32)	1164(4.66)	1557(5.2)	1618(5.57)	1886(5.61)	1167(5.86)	54.816	< 0.001
**16**	5783	3.18(3.10–3.26)	229(2.61)	433(2.94)	606(2.93)	732(2.93)	890(2.97)	1103(3.79)	1142(3.39)	648(3.26)	31.667	< 0.001
**58**	5014	2.76(2.68–2.83)	203(2.32)	368(2.49)	536(2.59)	574(2.3)	788(2.63)	871(3)	1012(3.01)	662(3.33)	54.806	< 0.001
**53**	4236	2.33(2.26–2.40)	87(0.99)	304(2.06)	488(2.36)	514(2.06)	773(2.58)	748(2.57)	781(2.32)	541(2.72)	53.654	< 0.001
**51**	3696	2.03(1.97–2.10)	84(0.96)	283(1.92)	417(2.02)	510(2.04)	651(2.17)	691(2.38)	678(2.02)	382(1.92)	14.165	< 0.001
**68**	2516	1.38(1.33–1.44)	77(0.88)	185(1.25)	259(1.25)	346(1.39)	482(1.61)	470(1.62)	441(1.31)	256(1.29)	6.255	0.012
**56**	2403	1.32(1.27–1.37)	84(0.96)	188(1.27)	286(1.38)	288(1.15)	412(1.38)	426(1.47)	467(1.39)	252(1.27)	5.036	0.025
**59**	2284	1.26(1.21–1.31)	82(0.94)	143(0.97)	202(0.98)	259(1.04)	398(1.33)	455(1.57)	475(1.41)	270(1.36)	49.118	< 0.001
**18**	2213	1.22(1.17–1.27)	81(0.92)	156(1.06)	264(1.28)	284(1.14)	421(1.41)	392(1.35)	394(1.17)	221(1.11)	1.439	0.23
**33**	1830	1.01(0.96–1.05)	95(1.08)	143(0.97)	196(0.95)	234(0.94)	314(1.05)	373(1.28)	318(0.95)	157(0.79)	0.502	0.479
**66**	1769	0.97(0.93–1.02)	60(0.68)	126(0.85)	200(0.97)	245(0.98)	289(0.97)	303(1.04)	316(0.94)	230(1.16)	9.730	0.002
**39**	1607	0.88(0.84–0.93)	31(0.35)	125(0.85)	173(0.84)	203(0.81)	268(0.9)	294(1.01)	290(0.86)	223(1.12)	25.595	< 0.001
**31**	1111	0.61(0.58–0.65)	53(0.6)	94(0.64)	136(0.66)	186(0.74)	178(0.59)	180(0.62)	170(0.51)	114(0.57)	5.388	0.02
**35**	905	0.50(0.47–0.53)	38(0.43)	57(0.39)	96(0.46)	131(0.52)	160(0.53)	167(0.57)	173(0.51)	83(0.42)	1.144	0.285
**45**	578	0.32(0.29–0.34)	16(0.18)	33(0.22)	46(0.22)	75(0.3)	116(0.39)	133(0.46)	86(0.26)	73(0.37)	11.282	< 0.001
**73**	305	0.17(0.15–0.19)	7(0.08)	22(0.15)	45(0.22)	37(0.15)	58(0.19)	46(0.16)	58(0.17)	32(0.16)	0.313	0.576
**82**	282	0.16(0.14–0.17)	0(0)	16(0.11)	25(0.12)	38(0.15)	67(0.22)	46(0.16)	63(0.19)	27(0.14)	9.073	0.003
**81**	4629	2.55(2.48–2.62)	182(2.08)	356(2.41)	603(2.92)	564(2.26)	719(2.4)	740(2.55)	909(2.7)	556(2.79)	8.654	0.003
**42**	2271	1.25(1.20–1.30)	75(0.86)	184(1.25)	226(1.09)	301(1.21)	394(1.32)	381(1.31)	436(1.3)	274(1.38)	12.924	< 0.001
**43**	2205	1.21(1.16–1.26)	126(1.44)	213(1.44)	258(1.25)	281(1.13)	362(1.21)	377(1.3)	384(1.14)	204(1.03)	11.069	< 0.001
**6**	1707	0.94(0.90–0.98)	62(0.71)	157(1.06)	177(0.86)	246(0.99)	295(0.99)	336(1.16)	287(0.85)	147(0.74)	0.649	0.42
**11**	1034	0.57(0.53–0.60)	36(0.41)	94(0.64)	123(0.6)	109(0.44)	157(0.52)	219(0.75)	215(0.64)	81(0.41)	0.651	0.42
**83**	275	0.15(0.13–0.17)	9(0.1)	34(0.23)	43(0.21)	30(0.12)	50(0.17)	44(0.15)	46(0.14)	19(0.1)	5.400	0.02
**Total**	58,233	32.05(31.83–32.26)	2023(23.09)	4496(30.48)	6505(31.47)	7351(29.44)	9799(32.72)	10,413(35.83)	11,027(32.78)	6619(33.26)	314.671	< 0.001

**Table 4:** Prevalence of single, double, and multiple HPV infections of 23 genotypes

HPV genotype	Single infection		Double infections		Multiple infections		Total infection	
	Positive no	%(95%CI)	Positive no	%(95%CI)	Positive no	%(95%CI)	Positive no	%(95%CI)
**HR-HPV**								
**52**	5849	3.22(3.14–3.30)	2443	1.34(1.29–1.40)	1288	0.71(0.67–0.75)	9580	5.27(5.17–5.38)
**16**	3295	1.81(1.75–1.87)	1539	0.85(0.80–0.89)	949	0.52(0.49–0.56)	5783	3.18(3.10–3.26)
**58**	2834	1.56(1.50–1.62)	1324	0.73(0.69–0.77)	856	0.47(0.44–0.50)	5014	2.76(2.68–2.83)
**53**	2262	1.24(1.19–1.30)	1210	0.67(0.63–0.70)	764	0.42(0.39–0.45)	4236	2.33(2.26–2.40)
**51**	1933	1.06(1.02–1.11)	1053	0.58(0.54–0.61)	710	0.39(0.36–0.42)	3696	2.03(1.97–2.10)
**68**	1342	0.74(0.70–0.78)	706	0.39(0.36–0.42)	468	0.26(0.23–0.28)	2516	1.38(1.33–1.44)
**56**	1194	0.66(0.62–0.69)	672	0.37(0.34–0.40)	537	0.30(0.27–0.32)	2403	1.32(1.27–1.37)
**59**	1073	0.59(0.56–0.63)	704	0.39(0.36–0.42)	507	0.28(0.25–0.30)	2284	1.26(1.21–1.31)
**18**	1176	0.65(0.61–0.68)	596	0.33(0.30–0.35)	441	0.24(0.22–0.27)	2213	1.22(1.17–1.27)
**33**	891	0.49(0.46–0.52)	554	0.30(0.28–0.33)	385	0.21(0.19–0.23)	1830	1.01(0.96–1.05)
**66**	843	0.46(0.43–0.50)	521	0.29(0.26–0.31)	405	0.22(0.20–0.24)	1769	0.97(0.93–1.02)
**39**	777	0.43(0.40–0.46)	487	0.27(0.24–0.29)	343	0.19(0.17–0.21)	1607	0.88(0.84–0.93)
**31**	559	0.31(0.28–0.33)	322	0.18(0.16–0.20)	230	0.13(0.11–0.14)	1111	0.61(0.58–0.65)
**35**	393	0.22(0.19–0.24)	282	0.16(0.14–0.17)	230	0.13(0.11–0.14)	905	0.50(0.47–0.53)
**45**	288	0.16(0.14–0.18)	169	0.09(0.08–0.11)	121	0.07(0.05–0.08)	578	0.32(0.29–0.34)
**73**	121	0.07(0.05–0.08)	103	0.06(0.05–0.07)	81	0.04(0.03–0.05)	305	0.17(0.15–0.19)
**82**	138	0.08(0.06–0.09)	67	0.04(0.03–0.05)	77	0.04(0.03–0.05)	282	0.16(0.14–0.17)
**Total**	24,968	13.74(13.58–13.90)	12,752	7.02(6.90–7.14)	8392	4.62(4.52–4.71)	46,112	25.38(25.18–25.58)
**LR-HPV**								
**81**	2462	1.35(1.30–1.41)	1313	0.72(0.68–0.76)	854	0.47(0.44–0.50)	4629	2.55(2.48–2.62)
**42**	1042	0.57(0.54–0.61)	706	0.39(0.36–0.42)	523	0.29(0.26–0.31)	2271	1.25(1.20–1.30)
**43**	1073	0.59(0.56–0.63)	608	0.33(0.31–0.36)	524	0.29(0.26–0.31)	2205	1.21(1.16–1.26)
**6**	776	0.43(0.40–0.46)	488	0.27(0.24–0.29)	443	0.24(0.22–0.27)	1707	0.94(0.90–0.98)
**11**	541	0.30(0.27–0.32)	272	0.15(0.13–0.17)	221	0.12(0.11–0.14)	1034	0.57(0.53–0.60)
**83**	123	0.07(0.06–0.08)	79	0.04(0.03–0.05)	73	0.04(0.03–0.05)	275	0.15(0.13–0.17)
**Total**	6017	3.31(3.23–3.39)	3466	1.91(1.84–1.97)	2638	1.45(1.10–1.51)	12,121	6.67(6.56–6.79)

**Table 5:** The sequence of genotypes co-infected by the top 11 genotypes in 23 genotypes in 2013–2020

genotype	Total of coinfected genotype^*^	No. 1 (cases)	No. 2 (cases)	No. 3 (cases)	No. 4 (cases)	No. 5 (cases)	No. 6 (cases)	No. 7 (cases)	No. 8 (cases)	No. 9 (cases)	No. 10 (cases)	Total of No. 1 to No. 10 (%)	LR-HPVS of No. 1 to No. 10 (%)^**^
HPV52	5725	HPV16(613)	HPV53(552)	HPV81(548)	HPV58(539)	HPV51(456)	HPV42(333)	HPV56(300)	HPV68(294)	HPV59(266)	HPV43(251)	4152(72.52)	1132(27.26)
HPV16	4019	HPV52(613)	HPV58(343)	HPV81(305)	HPV53(294)	HPV51(269)	HPV33(210)	HPV43(200)	HPV18(196)	HPV56(186)	HPV59(183)	2799(69.64)	505(18.04)
HPV58	3516	HPV52(539)	HPV16(343)	HPV81(340)	HPV53(255)	HPV51(227)	HPV42(181)	HPV56(175)	HPV59(166)	HPV68(153)	HPV43(140)	2519(71.64)	661(26.24)
HPV81	3537	HPV52(548)	HPV58(340)	HPV16(305)	HPV53(282)	HPV51(269)	HPV68(192)	HPV43(184)	HPV42(178)	HPV56(175)	HPV59(162)	2635(74.50)	362(13.74)
HPV53	3244	HPV52(552)	HPV16(294)	HPV81(282)	HPV58(255)	HPV51(214)	HPV56(171)	HPV42(166)	HPV43(164)	HPV68(156)	HPV66(135)	2389(73.64)	612(25.62)
HPV51	2890	HPV52(456)	HPV16(269)	HPV81(269)	HPV58(227)	HPV53(214)	HPV42(147)	HPV59(146)	HPV68(129)	HPV18(128)	HPV6(125)	2110(73.01)	541(25.64)
HPV68	1937	HPV52(294)	HPV81(192)	HPV16(161)	HPV53(156)	HPV58(153)	HPV51(129)	HPV56(105)	HPV42(101)	HPV43(100)	HPV59(89)	1480(76.41)	393(26.55)
HPV56	2091	HPV52(300)	HPV16(186)	HPV58(175)	HPV81(175)	HPV53(171)	HPV51(117)	HPV68(105)	HPV43(101)	HPV59(94)	HPV18(91)	1515(72.45)	276(18.22)
HPV59	2098	HPV52(266)	HPV16(183)	HPV58(166)	HPV81(162)	HPV51(146)	HPV53(133)	HPV42(106)	HPV43(100)	HPV18(99)	HPV66(98)	1459(69.54)	368(25.22)
HPV42	2135	HPV52(333)	HPV58(181)	HPV16(178)	HPV81(178)	HPV53(166)	HPV51(147)	HPV43(110)	HPV59(106)	HPV68(101)	HPV56(89)	1589(74.43)	288(18.12)
HPV18	1804	HPV52(213)	HPV16(196)	HPV58(137)	HPV81(135)	HPV53(134)	HPV51(128)	HPV59(99)	HPV56(91)	HPV42(88)	HPV43(88)	1309(72.56)	311(23.76)

* means the total number of genotypes co-infected with the remaining 22 genotypes

** means the percentage of the sum number of LR- HPV infections among No. 1 to No. 10 and the total number of 10 genotypes among No. 1 to No. 10.

## Discussion

This study analyzed the prevalence and genotype distribution of HPV infection among women in Chengdu who came to CWCCH for gynecological examination and health examination.The data from five continents [24] showed HPV prevalences were remarkable differences, not only between regions but also between countries and among studies within the same region,and the estimated global HPV prevalence was 11.7%,and Sub-Saharan Africa (24.0%), Eastern Europe (21.4%), and Latin America (16.1%) showed the highest prevalences,and the most common HPV types found among 215,568 women with normal cytological findings worldwide were HPV types 16, 18, 52, 31, 58, 39, 51, and 56, and HPV-6 was the most frequent low-risk type in the Americas [24]. In this study, the HPV infection rate in Chengdu was 23.28%, which was within the scope of 37 cities [25] (18.42-31.94%) in China, but it was significantly higher than those of domestic developed citys Shanghai [26] (18.81%) and Guangzhou [27] (18.71%), and very similar to that of Zhejiang [28] (22.3%). This study revealed that the HPV infection rate among females was relatively high in Chengdu, it is an urgent to strengthen the HPV vaccination and standardize the high coverage of HPV screening to reduce the incidence of cervical cancer among wowen in Chengdu.

In this study, the top five genotypes (HPV52, 16, 58, 81, and 53) were different from those of in Shanghai [26] (HPV 52,16,58,53 and 61), Guangzhou [27] (HPV 52,16,58,53 and 81), Zhejiang [28] (HPV 52, 58, CP8304, 16 and 51) and 37 cities [25] (HPV16, 52, 6, 58 and 11). It was evident that HPV-52, -16, and − 58 exhibit higher infection rates among women in Chengdu and many cities in China [25–28], and HPV-52 and − 58 also demonstrate high infection rates in Asia [24]. The differences of the top five genotypes reflect regional differences in HPV genotypes, which are related to local health awareness, economic level, and health habits. When developing vaccines suitable for different geographical regions, regional variations in the distribution of certain HPV types should be taken into account [3]. The HPV genotype distribution in this study may provide guidance for the development and adoption of vaccines. The more HPV genotypes antigens a vaccine contains, the stronger its protective effect against diseases related to HPV genotypes. Currently, four vaccines containing HPV-52, and HPV-58 have been developed and undergone clinical trials in China (quadrivalent, hexavalent, nonavalent, and quadrivalent), with Shanghai Biotech Co., Ltd.‘s nonavalent vaccine entering Phase III clinical trials [19]. The existing nonavalent HPV vaccine prevents infections and diseases related to HPV-31, 33, 45, 52, and 58 in susceptible populations, and induces antibody responses against HPV-6, 11, 16, and 18 [29]. Considering that in the Chengdu region, the nonavalent HPV vaccine can prevent diseases caused by the first three HPV genotypes (52, 16, 58), it is far superior to the quadrivalent HPV vaccine, which only protects against genotypes 16, 18, 6, and 11. Therefore, among the currently available vaccines, the nonavalent HPV vaccine is more suitable for women in Chengdu.

In this study, the prevalences of HPV infections among females all showed a bimodal U-shaped distribution with age. The first peak occurred at ages group < 20 years (42.97%), decreased sharply after the first peak, and maintained a plateau at middle age (20–59 years), the second peak common appeared at 60–69 years group (37.56%), followed by a moderately decline, and reached lowest prevalence at year groups ≥ 70 (25.72%). In fact, HPV infection rates are highest in young women and a smaller second peak in older people, which were common in Shanghai [26] (< 24 years,55–64 years), Guangzhou [27] (< 21 years, > 60 years) and Zhejiang [28] (≤ 20 years, 61–70 years). The age distribution of cervical HPV infection showed a bimodal curve in half of five continental regions, with a first peak at younger ages (just after sexual debut), a lower prevalence plateau at middle ages, and a variable rebound at older ages (≥ 45 years) [24]. The first peak among young women (< 20 years) is generally attributed to higher levels of sexual activity with multiple partners and low viral immunity [30]. However, most HPV infections in young women that are accompanied by simultaneous epithelial dysplasia undergo spontaneous clearance under immunological surveillance within 1–2 years [6, 31–32], and only a small percentage of persistent infections that cannot be cleared progress to cervical cancer after 10 to 20 years. In order to prevent young women from continuing HPV infection and progressing into cervical cancer, HPV vaccination is a very cost-effectiveness for women beforer sexual debut,because the earlier the HPV vaccine is vaccinated, the higher the antibody titer, and the better the protective effect [33]. The protection rate of bivalent, quadrivalent and nonavalent vaccines against HPV16/18 induced cervical intraepithelial neoplasia grade 2 or worse could reach 98.0%~100.0%, and the quadrivalent vaccine can also prevent 90–100% of genital warts caused by HPV6/11, and the nonavalent vaccine not only has a pre-prevention effect that is not inferior to the quadrivalent vaccine, because it can also prevent about 97% of high-grade precancerous lesions and cancers of the cervix, vulva and vagina caused by HPV31/33/45/52/58, moreover HPV vaccination has a strong group effect or indirect protection against unvaccinated females [29, 34–38]. A national multi-center epidemiological survey [39] shows that the first sexual intercourse of young women in China generally occurs at the age of 17, thus Chinese scholars [39] recommended that the junior middle school girls (13 to 15 years) be the preferred groups for vaccination,and the Chinese government has adopted the recommendation, and the next step will be to make the HPV vaccine free for 13–15 year junior middle school students.

In this study,the reason why HPV infection rates remain low in middle age (30–49 years) may be due to increased autoimmune function and stable sexual partners in marriage. Considering that the prevalence of CIN high-grade lesions is highest at the age group 30–49 years, and screening and treatment of HPV-related high-grade lesions at this age group is very important to reduce the incidence and mortality of cervical cancer, hence the WHO [20] calls for women to be screened twice for HPV (once before age 35 and again before age 45), with 70% coverage. Twice in a life time screening between 30 and 49 years can be highly protective and is very cost-effective (With one or two screening strategies for HPV testing, the lifetime risk of cancer was reduced by about 25 to 36%, with a life cost of less than $500 per year). In particular, screening of women aged 30 to 49 years is focused on full coverage rather than the number of lifetime screenings [40].

For the second minor peak of infection, the higher rate of infection in old women (60–69 years) may be due to immune disorders during the menopausal transition, inability to clear and suppress the virus effectively, as well as the persistence of the virus or the reactivation of latent HPV [41]. In older women (60–69 years) HPV screening and cytological tests are also necessary due to a small peak of infection, and screening should be continued over 65 years old among women who have never been screened in the past 10 years. For women over 65 years old, we should note that the prevalence rate for those aged ≥ 70 (23.91%) was higher than the overall prevalence rate (23.28%). This could be attributed to the fact that women at this age group usually do not visit hospitals unless they have HPV-related lesions such as genital warts or cervical lesions, or unless they are actively screened for HPV infection due to a diagnosis by a doctor.

In terms of the infection patterns,as the multiplicity of HPV infections increases, the infection rate decreases from 2013 to 2020. HPV single infection (73.26%) of females was the dominant pattern, followed by double infection (19.17%). Regarding the causes of multiple infections, some scholars [42] have proposed that it is determined by the immune mechanism. The potential impact of multiple infections on cervical cancer risk remains debatable [42–45], with one side arguing that women with multiple infections were at significantly higher risk of cervical disease than women with single infections, but multiple infections followed an independent process leading to disease [42]. The risk of cervical cancer was 31.8 times higher with multiple infection and 19.9 times higher with single HPV type [43]. Multiple HPV infection was an important risk factor for CIN ≥ 2 [44]. The other side [45] pointed out that there was no correlation between multiple infection and the severity of the lesion, but only he HPV subtype infected. In either case, HPV multiple infections are more serious than no infection, suggesting the importance of vaccines and screening.

Among 42,297 HPV positive women, HR- and LR- HPV coinfections accounted for 13.59% and 43.68% in HR- HPV infections and in LR-HPV infections,respectively. Thus, it means that 13.59-43.68% of patients with genital warts are infected with HR- and LR-HPV coinfections, therefore, patients with genital warts may have HR-HPV lesions, and when treating genitals, it is necessary to screen for HR-HPV infection and conduct appropriately treatment. As can be seen from Fig. 3, infection rates of HPV6 and HPV81 ranked second at the age < 20 and 40–59 groups, respectively, and the incidence of genital warts may be greater at this age, which is worth noting. HPV16 ranked first at the age group ≥ 70 years old, which may be related to the low clearance rate of infection at this age group.

The study has several limitations. First of all, this study is a hospital-based survey, and the population is limited, including a part of gynecological patients and a part of physical examination women, which does not cover the whole female population in Chengdu, so it cannot represent the general population of Chengdu. Second, it lacks cellular or pathological data from positive cases, making comparative statistics infeasible. Third, women’s vaccination rates were not considered, so the specific impact of the vaccine on HPV infection rates could not be calculated. Fourth, the lack of information on lifestyle habits, reproductive history, socioeconomic status, and sexual behavior makes it difficult to provide practical guidance on the prevention of HPV infection.

## Conclusions

In conclusion, The prevalence of HPV infection among women in Chengdu is higher than domestic certain developed citys.The top 5 genotypes of HPV infection among females in Chengdu were HPV52, 16, 58, 81 and 53, therefore,among the five vaccines currently available, the nine-valent HPV vaccine was more suitable for Chengdu women. Age-specific HPV infection showed a U-shaped bimodal pattern, with two peaks at age < 20 and 60–69. Considering the first peak (< 20 years), HPV vaccination should be prioritized among junior high school girls (13 ~ 15 years old), and despite a plateau at middle age (20–59 years), screening and treatment of HPV-associated high-grade lesions at the age group 30–49 would be important, a second peak occured in older women (60–69 years), and HPV screening and cytology tests should not be lacking. For patients with genital warts, it is necessary to screen for high-risk HPV infection and provide appropriate management and treatment. Given the limitations of this study, future HPV research should aim to achieve full coverage of the target population, and our studies should also include cellular or pathological data of HPV-positive cases, vaccination rates, and various lifestyle details.

## Data Availability

No datasets were generated or analysed during the current study.

## Abbreviations

HPV: Human papillomavirus
HR: High-risk
LR: Low-risk
CWCCH: Chengdu Women’s and Children’s Central Hospital
95% CI: 95% confdence interval
VLP: Virus-like particle
